# TORCH (Toxoplasmosis, Other, Rubella, Cytomegalovirus, Herpes Simplex Virus) Infection and the Enigma of Anomalous Fetal Development: Pregnancy Puzzles

**DOI:** 10.7759/cureus.51534

**Published:** 2024-01-02

**Authors:** Nainita Patel, Kamlesh Chaudhari, Dharmesh J Patel, Jalormy S Joshi, Garapati Jyotsna

**Affiliations:** 1 Obstetrics and Gynaecology, Jawaharlal Nehru Medical College, Datta Meghe Institute of Higher Education and Research, Wardha, IND

**Keywords:** antenatal care, pregnancy complications, anomalous fetal development, cmv infection, torch infection

## Abstract

The cytomegalovirus (CMV), a common DNA virus with a high global seroprevalence, is the primary cause of teratogenic congenital infections, which presents a serious risk to public health. Maternal CMV infection is linked to congenital CMV (cCMV), a major contributor to non-genetic sensorineural hearing loss, cognitive developmental impairments, and cerebral palsy in infants. Transmission might occur through direct contact with infected bodily fluids, with higher transmission rates after primary infection and an increased risk of severe fetal effects before 20 weeks. The mother and fetus do not get immunity from a prior infection. Fetal growth restriction, fetal loss, and cerebral or extra-cerebral abnormalities that can be detected by ultrasonography are possible presentations of cCMV. Specific antibody detection or seroconversion is required for the diagnosis of maternal CMV during pregnancy. Amniocentesis is used to diagnose fetal CMV during pregnancy after eight weeks of presumed maternal infection and 17 weeks of gestation. The main preventive measure is hygiene education, as the effectiveness of immunoglobulins, antiviral medications, and vaccines is still up for debate. The focus is particularly directed toward the anomalous fetal outcomes observed during the course of the pregnancy.

## Introduction

*Toxoplasma gondii*, *Rubella* virus, *Cytomegalovirus* and *Herpes simplex* virus constituting the TORCH complex are responsible for adverse fetal outcomes, miscarriages, and congenital defects. Around the world, TORCH infections are reported to have a significant effect on maternal and neonatal health [1]. A research study of women with bad obstetric history and seroprevalence of TORCH infections reported an IgM seropositivity of 3.41%-22.64% and IgG seropositivity from 19.2% to 70.51% for TORCH agents. TORCH seropositive women had congenital anomalies such as cardiac malformations, hydrocephalus, cataracts, and congenital rubella syndrome [2]. Congenital cytomegalovirus (cCMV) infection is recognized as a public health concern. However, the availability of recommendations and guidelines for the management of cCMV infection is limited. Despite the establishment of an informal International Congenital Cytomegalovirus Recommendation Group in 2015, which recently issued consensus recommendations for preventing and diagnosing maternal cytomegalovirus (CMV) primary infection during pregnancy, as well as for diagnosing and treating cCMV in neonates [3], there remains a shortage of comprehensive guidance. Presently, within France, conflicting national recommendations on systematic screening for CMV during pregnancy and at birth are evident, varying among different medical societies, including the Academie de Medecine, Collège National des Gynecologues Obstetriciens de France (CNGOF), and Haut Conseil de Sante Publique (HCSP) [4-6]. On a broader scale, the management of maternal CMV infection continues to pose challenges in numerous countries. While present in the maternal serum, CMV infiltrates and traverses the placenta, displaying a preference for various cell types in the fetal brain, such as astrocytes and neural stem cells. Following infection, these cells become conducive to continued viral replication within the brain, leading to consequential damage to the central nervous system. CMV-induced hearing loss is believed to stem from viral labyrinthitis. The predilection of CMV for cells within the central nervous system and the inner ear significantly contributes to the clinical manifestations of cCMV, encompassing brain abnormalities, hearing loss, seizures, microcephaly, and intrauterine growth restriction [7]. This case report aims to add to the developing knowledge database in maternal-fetal medicine, shedding light on the clinical intricacies and potential strategies for the management of pregnant individuals facing TORCH infections, with a particular focus on the profound implications of CMV. Through this exploration, we seek to enhance our understanding of the challenges posed by TORCH infections during pregnancy and to advocate for tailored and informed approaches to optimize maternal and neonatal outcomes.

## Case presentation

**Figure 1:**
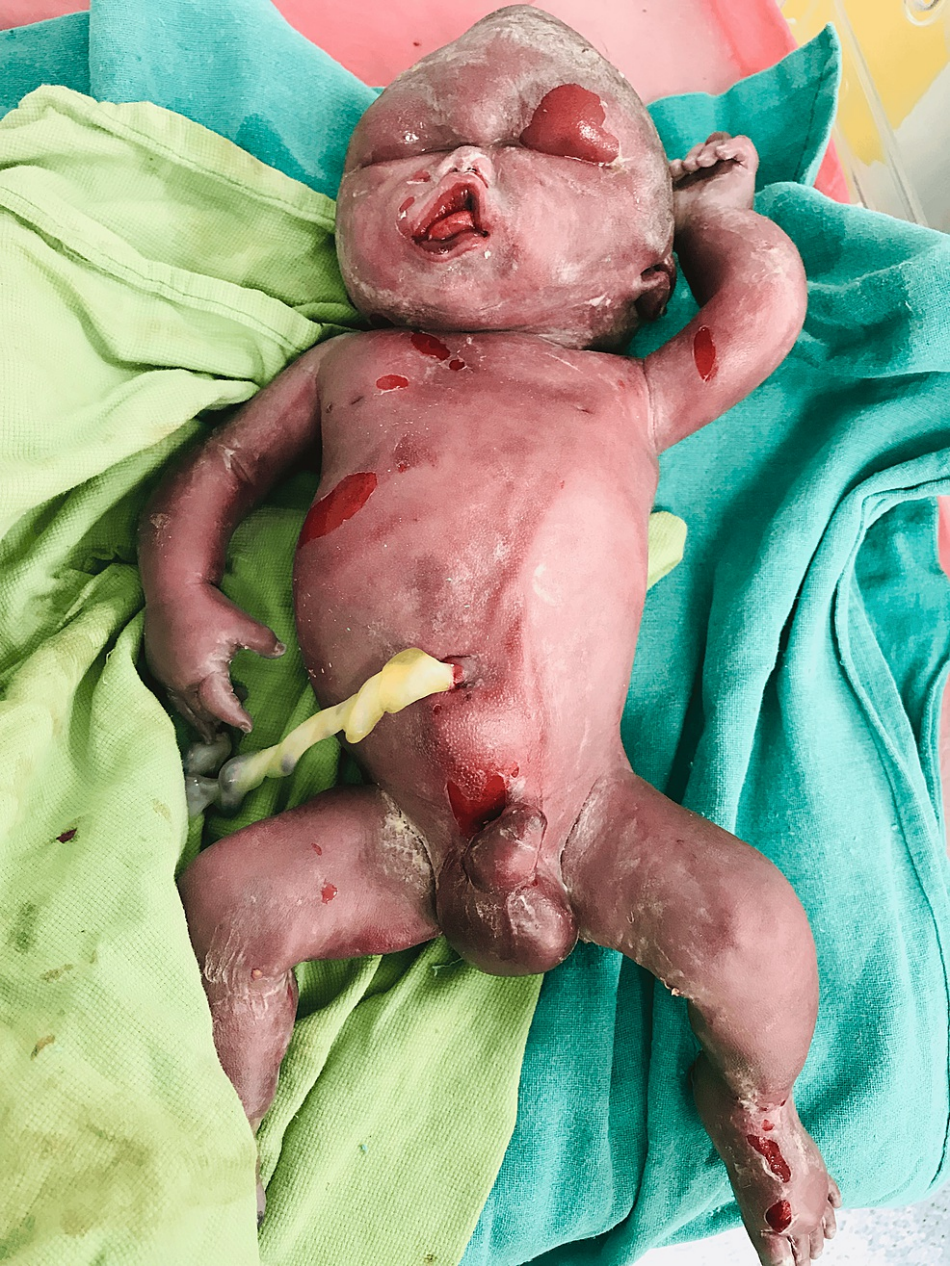
Male infant born with anomaly

A 26-year-old female patient with 29 weeks' gestation in view of pre-term labor pain presented to our tertiary care center. The patient was admitted with chief complaints of recurrent abdominal pain and breathlessness. The patient did not have a history of any significant chronic illness. The patient had a history of two spontaneous abortions in the past at approximately four months of gestation each. The blood group of the patient and his husband was B-RhD positive (B+). The patient was also subjected to chromosomal analysis in a previous pregnancy which was suggestive of monosomy of chromosome 15 (45-0-0). The patient had a positive TORCH test (Rubella IgG + CMV IgG) which was reported at five months of gestation in the current pregnancy. At admission, P/A showed uterus term size with the variable position of the fetus and more than adequate liquor. The patient was managed on Tab. Torchnil BD x 21 days/ week. Patients’ vitals on admission were BP 110/80 mmHg, RR-16/ min, pulse 76/min, and normal temperature. The ultrasound report confirmed polyhydramnios. This was managed by amnioreduction (thrice) to relieve polyhydramnios symptoms and the pregnancy was tried to be preserved until lung maturity of the fetus. The patient underwent the dual marker test at 13 weeks of gestation for the current pregnancy, which resulted in a low risk of trisomy for chromosome 13, 18, and 21. The patient was also scanned for a nuchal translucency scan for trisomy for Downs syndrome (chromosomal trisomy 21), Patau syndrome (chromosomal trisomy 13), and Edwards syndrome (chromosomal trisomy 18), which revealed an increased NT of 2.5 mm and anomaly scan had nuchal fold thickness of 7.2 mm, and the nasal bone was 45 mm. Intracardiac echogenic foci were 2.7mm in the left ventricle and right ventricle which was equivocal; hypoplastic, diffuse subcutaneous edema in the head, neck, and face with bilateral pleural effusion was observed. Ascites was present, with an abnormal thorax, and absent stomach bubble and more than adequate liquor (>25 LI) were noted in the fetal anomaly scan. Based on these abnormalities, the patient was further advised to go for a Quadruple marker test and also suggested medical termination of the pregnancy. However, the patient was not willing and wanted to continue the pregnancy. The patient had a spontaneous rupture of the membranes with fetal distress at 31 weeks' gestation. Fetoplacental insufficiency and absent end diastolic flow in the umbilical artery with thick MSL with pathological CTG resulted in a decision for EMLSCS and the patient was operated on. The fetal heart rate was not localized in USG and a spalding sign was seen in USG. A male baby weighing three kilograms, the baby did not cry at birth and the APGAR score was 0/10 and the baby had intrauterine death (Figure 1).

The patient recovered well. The parents were counseled for genetic testing of the dead fetus, but they opted not to. 

## Discussion

TORCH infection is the known cause of perinatal morbidities and mortalities. TORCH infections in females of childbearing age are a prime concern around the world. When these infections occur during the course of pregnancy, they can risk the life of the fetus largely and also decrease survival rates due to increased risk of associated morbidities. TORCH infections have also been associated with acquired congenital disease worldwide with infants affected with rates as high as 800/100000 live births (CMV) in developed countries such as the United States [8]. Most of the cases where mothers were TORCH infection positive in the first trimester of their pregnancy resulted in miscarriages and high fetal mortality rates. Also, stillbirths and congenital anomalies were common in such females. Morbidities such as congenital heart defects, hydrocephalus, splenomegaly, hepatomegaly, jaundice, fever, and rashes are commonly reported in infants born TORCH-positive pregnant females [9]. Research evidence presents that infections gained during pregnancy or neonates have been a major contributor to morbidities in newborns. A bad obstetric history has also been observed when a developing fetus acquires TORCH infection which results in miscarriages. Pregnancy outcomes in females are positive to TORCH agents and are usually observed as negative as miscarriages, improper fetal development, pre-term delivery, stillbirth, and/ or other pregnancy-related complications [10]. Severities and morbidities in fetuses depend on the degree and number of agents causing the infection. Each agent of the TORCH complex (Toxoplasmosis, Other agents, Rubella, Cytomegalovirus, and Herpes Simplex virus) affects the developing fetus and varies the normal developmental path. Primary infection in pregnant mothers gives birth to cCMV-infected neonates. Congenital CMV infections mostly lead to neurological damage such as mental retardation, visual impairment, hearing loss, and others. Still birth and mortality rates are also higher in CMV-infected pregnant mothers [11]. Fetal screening through various means as blood tests, amniotic fluid tests, and viral cultures accompanied by radiological tests for physiological defects are recommended, to confirm the fetal infections [12]. Based on these results, a comprehensive management approach is recommended by a team of clinicians from different specialties and the decision to continue/ terminate the pregnancy can be made. Currently, the provision of antenatal education regarding the risks associated with cCMV is not widely integrated into the standard practices of many obstetrician-gynecologists and midwives in India. This observed gap in educational practices may be attributed to the uncertainties surrounding antenatal screening and treatment for cCMV, contributing to a lack of consensus in the medical community. The prevalence of various misconceptions related to cCMV is outlined in Table 1.

**Table 1:** Misconceptions and facts about congenital cytomegalovirus cCMV: Congenital cytomegalovirus infection; IgG: Immunoglobulin G; TORCH: Toxoplasmosis, Other, Rubella, Cytomegalovirus, Herpes Simplex Virus; CMV:  cytomegalovirus Data from [13]

Misconception	Fact
cCMV is uncommon.	It is the most prevalent TORCH infection at an incidence of 1 in every 200 live births.
cCMV is unpreventable.	Infection risk is lowered by frequent hand washing and avoiding small children’s saliva.
A woman is immune if she has a past history of CMV.	Even if a person has previously acquired IgG antibodies, they may reactivate a latent infection or contract a new strain of CMV while pregnant.
A neonate won't experience any aftereffects if they don't exhibit any cCMV symptoms at birth.	Babies who have cCMV but do not exhibit any symptoms at birth (referred to as asymptomatic cCMV) might still experience hearing impairment at birth and are susceptible to hearing impairment that develops later in life.
There is no treatment for neonates with asymptomatic cCMV.	Early intervention services and careful observation of vision, development and hearing can be a part of the treatment plan, as antiviral medications have not yet been marked risk-free in asymptomatic neonates.
Pediatricians diagnose the majority of newborns with cCMV at birth.	At birth, cCMV is misdiagnosed in over 90% of episodes. The majority of cCMV cases exhibit weak or nonexistent symptoms, making the diagnosis tricky.

These facts further contribute to the persistence of a tendency to underestimate the potential risks posed by this virus. This lack of awareness underscores the need for a more comprehensive understanding of cCMV and its associated challenges within the Indian obstetric care context. Transmission of CMV occurs through bodily fluids, modifying antenatal behaviors can possibly lessen the chance of getting infected. Avoiding oral contact with the body fluids of young children, who have been reported as potential carriers of the virus might help in infection risk reduction. Common advised behavioral changes include avoiding sharing of food and utensils, frequent hand washing after diaper care, avoiding kissing young children, and preventing any type of contact with body fluids of children. It is important to note that, similar to many infectious diseases without available vaccines, behavioral adjustments cannot entirely eliminate the risk of cCMV. Nonetheless, emerging evidence suggests that providing antenatal education on cCMV and strategies to reduce risk may significantly decrease the incidence of antepartum seroconversion. Some professional organizations advocate for regular counseling on strategies to reduce CMV risk during preconception and prenatal care. However, not all organizations share this recommendation. The 2015 Practice Bulletin on CMV infection during pregnancy from the American College of Obstetricians and Gynecologists characterized antenatal CMV patient education as “unproven as a method to reduce the risk of cCMV infection”. Additionally, the bulletin suggested that implementing behavioral modifications may be deemed impractical or burdensome [14]. Serological testing of CMV IgG and IgM is done for diagnosis of recent CMV infection. But routine antenatal checkup for CMV infection is controversial because in the absence of symptoms, interpretation of CMV IgG and IgM tests can be very challenging. Positive IgG suggests prior life exposure to CMV and positive IgM suggests acute infection but cannot determine how recently. Specialists in maternal and fetal medicine determine the course of treatment since there are currently no guidelines for tracking and treating cCMV during pregnancy. The use of antiviral medication for antenatal cCMV treatment is not advised due to inconclusive evidence and potential side effects. Valaciclovir has been shown in small studies to reduce vertical transmission by 60%, but large randomized controlled studies have not confirmed its efficacy. Furthermore, research has shown that CMV hyperimmunoglobulin does not improve fetal outcomes or risk reduction of vertical transmission. Hence, it is not recommended. Although there is no evidence supporting an increased risk of premature rupture of membranes in CMV-affected pregnancies, some studies have reported a higher prevalence of cCMV in preterm neonates [3].

## Conclusions

This case report illuminates the intricate landscape of TORCH infections during pregnancy, with a specific emphasis on the challenges posed by cCMV. The lack of routine antenatal education and clearer guidelines with educational initiatives are imperative to equip healthcare providers, particularly obstetrician-gynecologists and midwives, with the knowledge needed for informed decision-making in managing TORCH infections. This case report highlights the importance of early diagnosis, pre-conceptional counseling, and management of TORCH infection in pregnancy with a focus to report outcomes that can improve medical knowledge for TORCH infection in pregnancy and will help obstetricians and gynecologists to treat CMV effectively.
